# Internet of Things and Machine Learning for Healthy Ageing: Identifying the Early Signs of Dementia ^†^

**DOI:** 10.3390/s20216031

**Published:** 2020-10-23

**Authors:** Farhad Ahamed, Seyed Shahrestani, Hon Cheung

**Affiliations:** School of Computer, Data and Mathematical Sciences, Western Sydney University, Second Ave, Kingswood 2747, NSW, Australia; s.shahrestani@westernsydney.edu.au (S.S.); h.cheung@westernsydney.edu.au (H.C.)

**Keywords:** dementia, internet of things, machine learning, IoT in healthcare, IoT in dementia care, dementia and smart environment

## Abstract

Identifying the symptoms of the early stages of dementia is a difficult task, particularly for older adults living in residential care. Internet of Things (IoT) and smart environments can assist with the early detection of dementia, by nonintrusive monitoring of the daily activities of the older adults. In this work, we focus on the daily life activities of adults in a smart home setting to discover their potential cognitive anomalies using a public dataset. After analysing the dataset, extracting the features, and selecting distinctive features based on dynamic ranking, a classification model is built. We compare and contrast several machine learning approaches for developing a reliable and efficient model to identify the cognitive status of monitored adults. Using our predictive model and our approach of distinctive feature selection, we have achieved 90.74% accuracy in detecting the onset of dementia.

## 1. Introduction

Self-management and self-dependent living in a smart environment have become a mere benefit for older adults in the modern socio-economic system. The older adults opted to live independently in their residences, instead of moving into aged care facilities. Therefore, many older adults live by themselves with minimum care in retirement villages. Often the older adults and their caregivers do not notice any cognitive decline to them as it develops slowly over time and become a norm in daily life. IoT sensor-based smart environment can assist in tracking the daily activities of the older adults and may suggest cognitive decline within the subject. Hence, older adults or caregivers can be alerted, and it can recommend visiting a geriatrician. Thus, IoT driven remote monitoring can assist older adults as a companion to advise on the possible symptoms of dementia and encourage them to visit a clinician. Additionally, this smart monitoring can enhance the peace of mind of older adults and caregivers in terms of smarter living.

There are various approaches to identify the onset of dementia by passive monitoring. There has also been some work to predict or detect dementia from the MRI scans and EEG dataset of the patients using image-based deep learning techniques [1,2,3,4]. Additionally, there have been some attempts to identify the onset of dementia from the behaviour based symptoms of older adults based on detecting wandering using GPS and accelerometer [5,6,7]. In the previous work, the authors have proposed a conceptual framework to identify early signs of dementia in older adults by remote monitoring [8]. They have provided an architectural view of how to detect the anomaly and the symptoms of the onset of dementia in older adults from various activities of daily life. Additionally, they have proposed a mathematical process to simplify the detection of the onset of dementia. Subsequently, this work has focused on developing a practical model for identifying the onset of dementia based on a publicly available dataset. The dataset contains information of the selected instrumental activities of the daily life of the participants. The authors have extracted and analysed the features from the dataset to develop models. These models consider the movements, and duration of selected tasks of the users in identifying cognitive impairments related to confusion, restlessness, forgetfulness and other behaviour of dementia patients.

The rest of the section discusses the characteristics of dementia and how IoT-based systems can monitor such characteristics, enabling early identification of the behaviour that may be related to cognitive impairments.

### 1.1. Characteristics and Adverse Effects of Dementia

**Figure 1:**
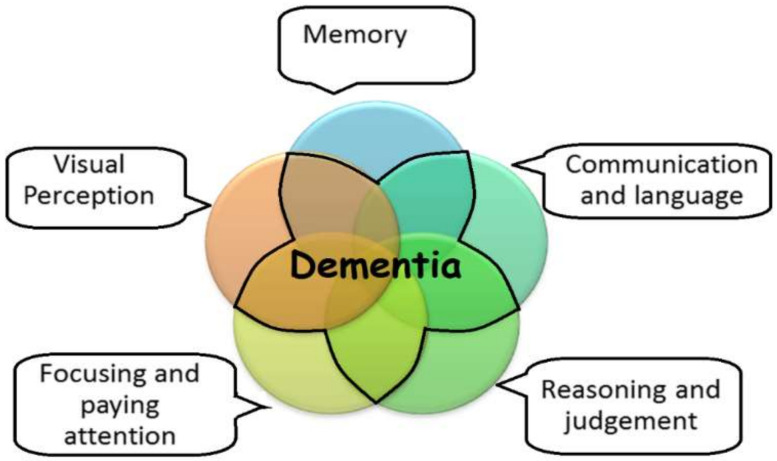
Five core symptomatic areas linked to dementia.

Dementia is a common phenomenon among older adults and one of the leading causes of death to the people who are over seventy years of old [9]. Degeneration of the cerebral cortex, the part of the brain responsible for thoughts, memories, actions, and personality, usually causes dementia [10]. Dementia patients suffer from the death of brain cells in a particular region. These dead cells lead to the cognitive impairments that characterise dementia. The well-being of the aging population is a significant concern in many parts of the world. Remote monitoring of the health and well-being of older adults is beneficial for their caregivers and their concerned relatives. Alzheimer Association has identified five core symptomatic areas that can be linked to dementia [11]. These are shown in Figure 1. Furthermore, these five types of core symptoms of dementia can be further sub-categorised into sub-symptoms [8].

In addition to these core symptomatic areas, Behavioural and Psychological Symptoms of Dementia (BPSD) can provide different indicators for detecting the onset of dementia. Some of those sub-symptoms of dementia can closely fit into BPSD signs. BPSD is a heterogeneous group of clinical phenomena. Around 80–90% of people with dementia have BPSD signs [12]. BPSD phenomena include disturbed emotions, altered mood, changes in perception and thought, unusual motor activity, deregulated sleep and wake up patterns, and personality changes [12].

The list below shows the common BPSD signs in dementia patients.

1. Anxiety
2. Depression
3. Hallucinations
4. Delusions
5. Psychomotor agitation
6. Aggression
7. Wandering
8. Screaming
9. Shouting
10. Biting
11. Spitting
12. Sexually inappropriate behaviour
13. Sleep disturbances
14. Personality change
15. Repetitive vocalisation
16. Apathy

The activities in the dataset have a correspondence with the symptomatic areas, as well as with the BPSD domains. As such, IoT-based systems can be used to identify the presence of the signs that may indicate a cognitive decline in an older adult, leading to the detection of the onset of dementia.

### 1.2. IoT and Continuous Monitoring

The advancement of small-scale inter-connected devices, namely, the IoT, provides an opportunity to monitor the well-being of the aging population in a smart home environment. Predicting the symptoms of the onset of dementia is a complex diagnosis process in medical science [13]. To detect the onset of dementia using remote monitoring, it is essential to monitor the Activities of Daily Life (ADL) of older adults. IoT devices and platforms can play a significant role in gathering users’ daily activities. Specific IoT sensors can discover some of the behaviours of older adults. For example, a motion sensor can detect movements in stairways, the lobby, and other parts of the house. Pacing and restlessness can be detected at night by motion sensors, which can indicate the wandering behaviour. Camera input for image processing can suggest and confirm various types of daily activities in the home settings. IoT smart power sockets can indicate the operational activity of any electronic devices; therefore, a possible daily activity can be registered. Additionally, there are some promising results to classify and identify cognitive impairment scenarios using image-based food stock monitoring in the refrigerator and to detect wandering using body-worn tracking devices. Hence, remote monitoring is useful in tracking the daily activities that will lead to the detection of cognitive decline and onset of dementia.

The contributions of this paper are the development of a model to identify the onset of dementia in a controlled residential setting. The previous work from the selected dataset only focused on identifying the person who has dementia [14]. However, our model is more flexible in identifying the onset of dementia by including the data from people with mild cognitive impairments. Additionally, this work takes a different approach to select features from the dataset and provides a better model with a lower number of features, comparing 38 features in this work by 352 features in the original work. Finally, a strong relation with Instrumental ADL (IADL) and the model is created to generalise the model for other datasets and living settings. Because the smart living environment can vary in different places; however, the IADL based model will be common for different living settings. The model is developed by utilising the classifier algorithms, neural networks, and supervised machine learning. Furthermore, this paper identifies the critical features within the activity data that are more valuable in detecting the onset of dementia through Machine Learning and the IoT.

The rest of the paper is organised as follows. Section 2 describes the related works and establishes the motivations of this work. Section 3 describes the methods used in this research to identify the onset of dementia from the behaviour of older adults. The dataset is described in detail in Section 4, including steps of data pre-processing and feature extraction methods. Section 5 discusses the feature analysis process, and the formulation of the machine learning model to identify the cognitive disorders of the elderly. This section also highlights the ranking of the features that are used for the model. Section 6 analyses the model training and testing performance. Concluding remarks and the direction of the future work in this domain from this research perspective is presented in Section 7.

## 2. Background and Related Works

IoT devices and platforms can play an essential role in accumulating data from the daily activities of the users and recognise daily [15,16,17]. Preetoom et al. have identified five main types of monitoring technologies intending to improve independent living [18]. Their study demonstrates the potential of using remote monitoring to improve the independent living of elderly persons in a pervasive smart environment. In the following sub-sections of this section, various methods used by the research community is introduced to identify the onset of dementia and cognitive decline.

### 2.1. Wearable Sensor-Based Monitoring to Identify the Onset of Cognitive Anomaly

Wearable sensor-based monitoring and analysis are gaining attention due to the availability of low power wearable devices. Using wearable sensors, the researchers of Parkinson@Home study group examined the feasibility and compliance to collect clinically relevant data for Parkinson disease. They have also tried to evaluate the usability of these data for answering clinical research questions as well as building a dataset for future research [19]. Wandering behaviour detection can also assist in identifying the symptoms of the early stage of Dementia. Kim and Lin et al. used Global Positioning System (GPS) and Radio Frequency Identification (RFID) based devices to detect wandering behaviour [5,6], which includes movement and travel patterns of the older adults in a smart environment.

### 2.2. Remote Monitoring to Identify the Onset of Cognitive Anomaly

Surveillance and remote monitoring are used mostly in security facilities for providing a secure environment for older adults. Additionally, privacy-aware remote monitoring can provide the required information in collecting ADL of older adults. There are various detection methods for ADLs in a smart environment [20,21,22]. More common approaches are video image-based and wearable sensor-based activity and ADL recognition. There have been attempts in using a deep learning behaviour model to understand the daily activities of people living a smart home. Intille et al. created a smart home laboratory to record the daily activities of the inhabitants [23]. Some human activities are quite complicated to detect either because they are composed of many lower-level actions, or because these actions may be different for different people or at different times. However, remote monitoring can still be beneficial and efficient for older adults and caregivers to keep track of the onset of dementia. The dataset used in this work contains the type and action detail of the IADL.

### 2.3. Activity Simulation to Identify the Onset of Dementia

Another trend from the literature is developing a home activity simulation model to identify an anomaly in ADL and flag cognitive decline or dementia-like behaviour. AlBeiruti et al. have used Hidden Markov Models to build a behavioural model based on raw sensor data. They claimed that their resulting model could detect sudden and gradual abnormalities, in older adults’ behaviour, which may be considered an indicator of dementia using simple binary sensors [24]. Abe Yuki et al. used an anomaly-based ADL to detect early signs of dementia with a success rate of 50–75% [25]. One of the major limitations of these predictive models is that these are created based on simulated activities. Hence, these models should be tested in real-life environments to validate accuracy and applicability.

### 2.4. Machine Learning Approaches to Identify the Onset of Dementia

Machine learning is a statistical learning technique that can assist in building a model to the pursuit of drawing some outcome from observed values. When the inputs are given, a classification model will try to predict the output value. These output values are labels that can be applied to a dataset. For example, when testing in the dataset “healthy”, or “cognitive-impaired”. There are three major approaches to machine learning: supervised, unsupervised and reinforcement learning. In a supervised model, a training dataset which is labelled with the outcome is fed into the machine learning algorithm. That let the model knows what is, for example, the sign of “cognitive impairment”, and the model can optimise the predictive function during the training. Afterwards, the test data sample is compared with the developed model to determine if there is a “cognitive impaired” event. This type of learning falls under “Classification”.

Various machine learning approaches have been used to identify the onset of dementia. Identifying the ADL is the key to discover the cognitive anomaly within older adults. Hence, there is some research work focusing on defining human behaviour and predict their actions based on simulation, wearable sensors, remote monitoring using the electronic data of daily life. For example, NK Suryadevara et al. attempted to forecast the behaviour of aged people in a smart home environment [26]. Choi et al. tried to use deep learning to predict human behaviour for smart homes [27]. Deep learning techniques can be useful for video analysis to detect ADL and discover an anomaly in the routine [28,29]. As some dementia patients manifest signs of repetitive speech, this repetition can also be detected with the deep neural network [30]. Ishi et al. tested an IoT based system to detect the onset of dementia where seven types of anomalies were considered, and success rates were from 36% to 93.6% using these anomalies [31]. Enshaeifar et al. used a Hidden Markov Model method to achieve an 80% accuracy in predicting the symptoms of the onset of dementia based on data received from IoT based sensors [32]. Hence, machine learning is being used extensively to identify the onset of dementia in a diverse direction.

It is important to identify and rank the ADLs and the symptoms of the behaviour to relate them with the cognitive decline of an older adult. Because these would improve the accuracy to identify the symptoms, and it will also assist in recognising which activities are important to track from a remote monitoring perspective. Further research work needs to be done on identifying repetitive speech on pre-dementia patients. Furthermore, other machine learning methods should be explored to improve accuracy as well as generalising the identification of the onset of dementia. To address some of these gaps, a model is presented in the next section that will identify the onset of dementia based on the characteristics of the completed ADLs that are critical to complete for older adults. A comparative analysis is provided in the later sections to find the important features that will be considered to develop an intelligent model.

### 2.5. Machine Learning Algorithms to Build the Model

In this work, various machine learning approaches are used to reach the best model based on the data used in the experiment. The overview of some of these classification algorithms and concepts are briefly mentioned below.

**Decision Trees:** A decision tree is a mechanical way to decide by dividing the inputs into smaller decisions. [33]. Decision tree models are used for comparison in this work.

**Linear Discriminant Analysis (LDA)**

The objective of linear discriminant analysis is the classification of cognitive healthy and cognitively impaired patients from the observations in this work. This analysis aims to develop discriminant functions that are nothing but a linear combination of independent variables. These variables will discriminate between the categories of the dependent variable in a perfect manner [34].

**Logistic Regression**

The logistic regression models the probability of the events in bivariate mode, which are essentially occurring as a linear function of a set of dependent variables [35]. These dependent variables can be represented in the binary (0 or 1, true or false, yes or no) values. It means that the outcome could only be in either one form of two. This method is utilised in this work to find the probability of cognitive decline or cognitively healthy.

**Naive Bayes**

The Naive Bayes classifier is also evaluated for this work. This classifier is designed for use when predictors are independent of one another within each class. However, it appears to work well in practice even when that independence assumption is not valid [36].

**Feed-Forward neural network (FFNN)**

FFNN is also used to build a model in this work. FFNN is one of the simplest forms of Artificial Neural Network (ANN), where the data or the input travels in one direction [37]. The data passes through the input nodes and exit on the output nodes. This neural network may or may not have the hidden layers. In simple words, it has a front propagated wave and no backpropagation by using a classifying activation function.

**Support Vector Machine (SVM)**

SVMs are models for regression and classification tasks. SVM models have two particularly desirable features: robustness in the presence of noisy data and applicability to a variety of data configurations. At its core, a linear SVM model is a hyperplane separating two distinct classes of data (in the case of classification problems) [38]. This separation is done in such a way that the distance between the hyperplane and the nearest training data point (called the margin) is maximised. Vectors that lie on this margin are called support vectors. With the support vectors fixed, perturbations of vectors beyond the margin will not affect the model; this contributes to the model’s robustness. By substituting a kernel function for the usual inner product, one can approximate a large variety of decision boundaries in addition to linear hyperplanes.

**Ensemble RUSBoost**

RUSBoost is a new hybrid sampling algorithm which learns from skewed training data [39]. This algorithm provides a more straightforward and faster approach to combine boosting and data sampling. This algorithm is used to apply on the dataset and compare it with other relevant algorithms with testing data. This learning algorithm performs well on the imbalanced dataset.

**K-nearest neighbour (KNN)**

The KNN algorithm is based around the simple idea of predicting unknown values by matching them with the most similar known values [40]. It is a method for finding the K closest points to a given data point in terms of a given metric. Its input consists of data points as features from testing examples, and it looks for K closest points in the training set for each of the data points in the test set. The output of KNN depends on the type of task. For classification, the output is the majority vote of the classes of the k nearest data points. That is, the testing example gets assigned the most popular class from the nearest neighbours. Several variations of KNN is tested to find a suitable KNN model in this work.

**K-fold Cross Validation:**

Cross-validation is a technique that involves reserving a particular sample of a dataset on which the model is not trained. Later, the model will be tested on this sample before finalising it [41]. The model should be trained on a large portion of the dataset. Otherwise, there is a high possibility of failing to discover the underlying trend in the data. Furthermore, it can eventually result in a higher bias.

On the other hand, a good ratio of testing data points is also needed. It is because the fewer amount of data points can lead to a variance error while testing the effectiveness of the model. It is also required to iterate the training and testing process multiple times. The dataset should be split to the train and test distribution to validate the model’s effectiveness.

In this work, several classifiers are used to train and validate the system models and compare them to identify which model should perform well based on the available dataset. K-fold cross-validation is primarily used in applied machine learning to estimate the skill of a machine learning model on new data. After the model is trained with some sample data, in this method, a limited number of sample is used to estimate how the model is expected to perform in general.

K-fold validation is incredibly helpful where the dataset volume is not very big. In this paper, the validation technique involves randomly dividing the dataset into 5 to 10 groups or folds of approximately equal size of samples. The first fold is kept for testing, and the model is trained on k−1 folds. Due to the size of the data points, it will be appropriate to use simple classifiers such as Decision Trees, Discriminants, Logistic Regression, SVM, KNN, and Ensembles.

## 3. Scheme of Identifying the Onset of Dementia

Identifying the early stages of dementia is challenging as the symptoms manifest slowly for a patient, and people around them may not notice the changes. Hence, there is a growing interest in detecting stages of dementia from the MRI scanning that is also under development [1]. The most common clinical diagnosis to detect cognitive decline performed by geriatricians is the Mini-Mental State Exam (MMSE). For mild dementia, they perform Alzheimer’s Disease Assessment Scale-Cognitive (ADAS-Cog) [42]. If the MMSE score starts to decline, it may indicate the person is developing dementia. Many older adults will normally live without realising the deterioration of their cognitive state and, therefore, may not seek medical attention.

Ambient sensors can be used to monitor and collect data of activities related to the five major symptoms categories and BPSD signs of the patients, as mentioned earlier. By monitoring the daily movement and activities behaviour and routine tasks through IoT devices, intelligent models can be formed to detect the onset of dementia. Artificial Intelligence and Machine Learning techniques will aid in identifying the patterns in cognitive state. Wandering behaviour, restlessness within a residence will generate unusual sensor firing that can help to develop the intelligent model. Hence, movement identification using motion and presence sensors and activity identification and completeness is an important part of the input data in our method.

Furthermore, completing a task correctly and with less repetition will reduce sensor activation in residence comparing to agitation, wandering, and forgetfulness. Hence sensor activation counter is considered as a source of building the model. Completing the ADL is a good sign of well-being of an older adult. Frequent missing of the essential ADL will raise concern to the caregivers about their cognitive health. Hence, in this study, the machine learning method is employed to study the ADLs from the dataset. This method will assist in discovering the durations and completeness of each ADLs to identify the onset of dementia.

### 3.1. Identification Method of the Onset of Dementia with IoT

The cognitive impaired person will have difficulties in performing IADL [43]. These are activities related to independent living and are valuable for evaluating persons with early-stage disease, both to assess the level of disease and to determine the person’s ability to care for himself or herself. The smart environment will sense the events from the motion sensors, power usages sensors and other related sensors to detect that an activity is taking place. This will facilitate the identification of the activity. The activity start time and end time will be logged as well as each of the sensor firing events and duration will also be logged to collect the movement history of events.

**Figure 2:**
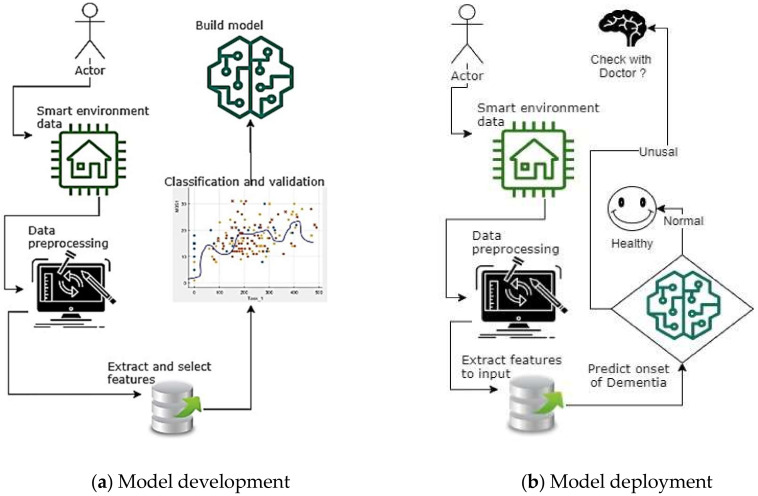
Flowchart of the system to identify the onset of dementia.

The architecture or components of the overall proposed system is shown in Figure 2. According to the flow, all the user activities will be recorded from the smart environment. These data will be pre-processed and filtered to prepare for extracting the features. Among the features, the critical one for this work is the sensor firing events, task completion time, and entry-exit sensors in between rooms and corridors. Furthermore, the total IADL activity completion counter and the duration to perform each IADL are also essential features that can be extracted from the IoT enabled smart environment. The features will be obtained and selected using the ranking process and analysing the importance and relevance to the application. Then the dataset will be ready to use in machine learning analysis to build a model.

In the next section, it has been discussed how to prepare the dataset and extract features to build the model.

## 4. Data Analysis and Feature Extraction

This work utilises the CASAS dataset [44]. This is a widely used dataset that has been developed by Washington State University. The dataset has low-level sensor information of ADL and IADLs of various participants. In this section, after presenting an overview of this dataset, our feature extraction method is described. The layout of the IoT sensors and corresponding framework is also narrated as part of dataset overview. A list of the IADL performed by the participants is also provided as part of the dataset overview. Later on, the data pre-processing steps and feature extraction process is elaborated in this section.

### 4.1. Dataset Overview

This dataset was collected from an IoT enabled smart environment. The participants were from various age groups, and the doctors diagnosed cognitive levels of the participants. The age range of the participants is mentioned in the dataset. The focus of this work was to extract information related to IADL from the dataset. In the dataset, the log files are named and numbered according to the participant’s serial number. The dataset also contains information about the cognitive state of the participant. Further details about the dataset are described in the following sections.

#### 4.1.1. The Layout of the IoT Devices

**Figure 3:**
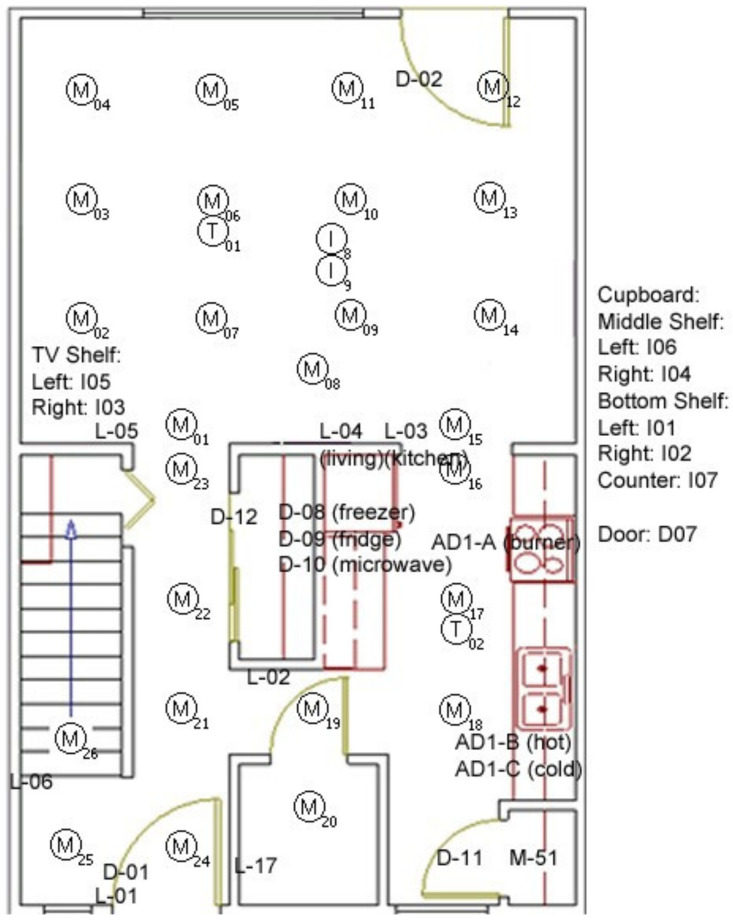
Layout of the IoT sensors in CASAS dataset.

The arrangement of the IoT sensors in the apartment is shown in Figure 3 [45]. The apartment has two floors. In this experiment on the data, only the eight ADLs are considered those that have been conducted on the ground floor. There is also additional data for few other activities that were also done on the second floor of the living space, which is not included in this study as the IADL are covered in the first major eight activities, as shown in Table 1. The ground floor has a living room, kitchen, change room, built-ins, and furniture. The doors have sensors attached to them. Motion sensors are mounted in various parts of the ceilings of the apartment, as per Figure 3. In Figure 3, the door sensors are marked as D0xx. The motion sensors are marked as M0xx, and Item sensors are marked as I0xx, the temperature sensor marked as T00x, Light sensor L0xx, and others.

There are motion sensors placed in between the corridors and room, such as M023, M001, M018, M021, M016, M015, and others, as shown in Figure 3. These are considered as transition sensors as they will capture the entry or exit of a room to another part of the apartment.

**Figure 4:**
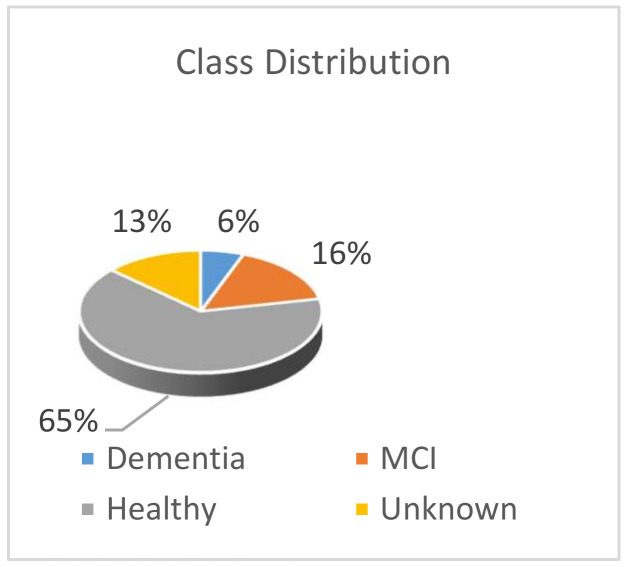
CASAS data class distribution of total participants.

This dataset contains the record of a total of 400 participants who performed the daily activities. However, 350 log files of the participants contain actual data, and the log files of 50 participants were not recorded in the database. From the available log files of the participants, over 35 features are extracted to develop the models. From these available log files, the participants belonged to one of two major cognitive groups: cognitively healthy and cognitive impaired. In the cognitive impaired group, there were a total of 20 participants of dementia patients and 56 participants of MCI patients. Some data points did not include the cognitive level of the patients; hence these are not used, for example, participants labelled as “other medical condition”, “at-risk” and “unknown”. The cognitive healthy data points were scattered into multiple age groups. Out of the cognitively healthy records, the middle age group (age range between 45–59) has 30 records. The young-old group age between 60–74 has 80 records. The 75+ years old cognitively healthy group has around 38 records. Furthermore, young adults who are cognitively healthy participants have 65 records. The overall data class distribution is provided in Figure 4.

In the dataset, there are 76 cognitive impaired class data and 228 cognitively healthy participants’ data. Here 46 records are representing an unknown state of the participant, so this portion of data is discarded for building the model as this will reduce the accuracy to identify someone having mild cognitive impairment to full dementia. The unknown class can be tested against the actual model to have a judgement on the performance of the model. A neurologist interviewed the participants and marked their cognitive levels. These assessments are used as ground truth for the classifier. The participants who were diagnosed with dementia met the Diagnostic and Statistical Manual of Mental Disorders (DSM-IV) criteria [46]. There were males and females among the participants. The participants were asked to perform selected IADLs in the smart home. While they complete activities, various sensors that were placed in the smart environment captured the progress of the activities to its completion [45].

#### 4.1.2. IoT Architecture of CASAS Environment

The physical layer of that system contains hardware components, including sensors and actuators. The architecture utilises a Zigbee wireless mesh that communicates directly with the hardware components. A publish/subscribe manager governs the middleware layer. The manager provides named broadcast channels that allow component bridges to publish and receive messages. Besides, the middleware provides valuable services, including adding timestamps to events, assigning unique identifiers, and maintaining a site-wide sensor state. The middleware layer of the CASAS data collection system also stores the sensor events to the database. The collected data is stored as per subject having their own data file. Each data file contains the timestamp of the events, sensor tag, and sensor state as below:

**09:07:02.27948 M015 OFF**
**09:07:03.6804 M013 ON**
**09:07:06.29772 M013 OFF**
**09:07:07.3707 D007 CLOSE**
**09:07:10.67025 M018 ON**
**09:07:10.90595 M019 ON**

As mentioned earlier, Mxxx represents the motion sensor number in the log file. Other sensors are abbreviated similarly. The dataset details can be found from the dataset source link provided earlier. After each of the events, a line or row is recorded into the log file, including the timestamp of the event. Besides, the sensor type and serial number are recorded with the sensor status (on/off/close).

#### 4.1.3. Activities of the Participants

The participants performed several activates. However, only the eight activities relate to IADL, which are interested in using to build the model, and these activities are mentioned in Table 1. These activities produced sensory events and the sequence of events as well. The values of these events are stored in the dataset. Participants were asked to perform the IADL activities in a sequence and within a specified time.

All the participants had a couple of hours to complete the requested task, and the activity log files were generated accordingly.

### 4.2. Data Preprocessing

The log files of 400 participants are processed to extract the data. Fifty of the log files did not have any relevant information, and they are excluded during the feature extraction process. The activity log files had thousands of timestamps with the sensor on/off, activity start/end logic embedded on it. Initially, there are ten categories of participants indicated into the original dataset, which were grouped into three parent categories for our data preparation process as the following.

- Cognitively Impaired
  ◦ Dementia
  ◦ MCI
- Healthy
  ◦ Middle age 45–59
  ◦ Young-old 60–74
  ◦ Old 75+
  ◦ Younger adults
  ◦ Younger adults, English second language
- Unknown
  ◦ Other medical
  ◦ At-risk—follow longitudinally
  ◦ Diagnosis not available

In the dataset, participants are labelled with a cognitive state with category number “1–10” based on the list above. The sensor activity logs are auto-generated based on the sequence of sensor activation. There was an activity observer who assisted in marking the log file to identify the beginning and end mark of a specified activity mentioned in Table 1. However, this can be automated through the activity recognition and identification using IoT that is discussed in a paper by the authors [8]. In the next section, it is described how the features are extracted from the log data files.

### 4.3. Feature Extraction from the Dataset

As mentioned in Section 3.1, the types of activities and parameters are relevant and essential to identify the onset of cognitive decline. Based on the information in the log files, all the available and extractable feature related to this work is considered. The duration of completing each IADL and ADL is an important feature that is possible to extract from the data. The motion sensor activation and deactivation events are also likely to collect that represent the activeness of a participant. Additionally, task completion and starting information are also possible to extract. For each participant, the total number of motion sensor firing for each sensor is taken as features. As there are 51 available motion sensors, each of them is counted as one of the features. Finally, a total of 59 features are extracted from the dataset. It is assumed that the duration of each task completion will be the most significant feature as the dementia patients should demonstrate difficulties in performing a task in the correct order and within an average period.

Consequently, the elapsed time to complete eight tasks is calculated as eight more feature inputs. Furthermore, the total activation count of the 51 motion sensors summed up to input as one more feature. Thus initially, a total of 59 features are extracted from the dataset. Our following algorithm is used to extract the features from the log files.

Algorithm 1 to extract features from the CASAS dataset:
**Algorithm 1** Procedure to extract features from the datasetStep 1. Start a loop up to the total number of data sample files. For each loop feature will be extracted and will be stored as a row of records. Step 2. Trim the loaded file in each loop to keep the relevant sensor data for the task 1 to 8. Step 2. Calculate the duration of each task (total of eight tasks) and store the record using an inner loop. Step 3. Counting sensor firing events for each relevant sensor (51 sensors) using an inner loop and store the record. Step 4. Repeat the steps (1 to 4) until all the data files are extracted for features. Export the output in a feature matrix table for Machine learning.

The source code for extracting the features, the dataset, and related codes are shared in the link [11]. The output file after running the data extraction is a feature matrix that has 51~59 features and 350 samples of the participants. The participants performed the tasks mentioned in Table 1 involves the sensors that are in Figure 3. The other irrelevant sensors, such as sensors number as M027 to M050, are removed from the feature matrix. These are irrelevant to build our model as these subsets of data are for other purposes, and these sensors are used for the upper floor as per the original dataset information. Finally, there are 35 features extracted and retained from the data log files. Additionally, based on these 35 features three more features can be generated as (1) “TotalTime” which is the sum of the total time for all the task, (2) “TaskScore” which counts how many tasks was completed and (3) “TotalEvents” which add up all the motion events of the relevant sensors to represent movements of participants.

Next, the dataset is divided into a training set with 56 instances of Dementia and MCI grouped into cognitive impaired and 194 instances of healthy adults. So, the training portion of the dataset has 250 unique data points. The test dataset contained 20 data points of cognitive impaired and 34 sets of healthy adults. During this data preparation process, it can be observed that the data is imbalanced, as shown in Figure 4. For experiment purposes, a second duplicate instance of train dataset was created to function as balanced data to train the FFNN model. A balanced dataset is required to avoid bias in developing an optimal FFNN model. Therefore, the second training instance data was balanced by duplicating the minor class data. Hence, the balanced train dataset contained 400 data points with duplicates in it.

Furthermore, the third duplicate of train dataset is created to balance the dataset using safe-level SMOTE algorithm [47,48]. This instance has a total of 391 data points where cognitive-impaired minority class is stretched to 197 records and remaining 194 records are for healthy older adults. All the developed models on the different training set will be tested against the same test dataset. There is a total of 38 features that are going to be evaluated to reduce dimension and sensor numbers for building a model. Some features will be dropped for model simplicity and efficiency. In the next section, the data analytics and feature selection process from the extracted data is discussed for that purpose.

## 5. Feature Analysis

In this section, the extracted features, selection process, and feature reduction process and outcomes are discussed. The features are extracted from the dataset to analyses the patterns within the classes. In Figure 4, it appears that this is an imbalanced dataset with a lower number of samples from demented and MCI patients who are grouped into the cognitive impaired person. Identifying a person with the early signs of dementia vs. healthy adults may require many sensors, processing, and costly smart environment. Therefore, the study of dimension reduction and ranking features of the dataset is important to reduce the number of required sensors to identify IADL. Hence, it is analysed in this section which features are more influential than the others and methods to rank them according to the experimental data.

### 5.1. Feature Data Preview

Before building the actual model, it is necessary to have a birds’ eye view of the data to discover inter-related parameters. The cognitive impaired person would have difficulties to complete the assigned tasks. Hence, they may miss out on some tasks. Even if they have completed a task, it is expected that it would require a longer time to complete it. Before any model is created, it is an initial expectation from the dataset. From the feature analysis point of view, these critical patterns should surface in the data. After careful examination, it is observed that some participants did not complete some tasks falling into either classification: cognitively impaired or cognitively healthy. For these undone tasks, the algorithm put straight ‘0 seconds’ to complete a specific task. These zeros represent missing values in the dataset. It would be rather a significant number which would represent not completion of a task. Hence, during data pre-processing, this Zero was replaced by a value twice the maximum value of that feature.

For data analytical purpose, this initial data is plotted with selected features to discover any exciting pattern to find the categories. The data is plotted in the parallel coordinate plot with Matlab^®^ using the standardisation plots, which provide the mean of each task completion time feature at zero and scales the predictors by their standard deviations. The standard deviation is calculated using the equation below,

(1) $$s\;=\;\sqrt{\frac{\sum {\left(X-\bar{X}\right)}^{2}}{n-1}}$$

where,

X = the features of each data point
*s* = sample standard deviation
∑ = sum of
$\bar{X}$ = sample mean
N = number of records (250 for imbalanced data and 400 for balanced data).

**Figure 5:**
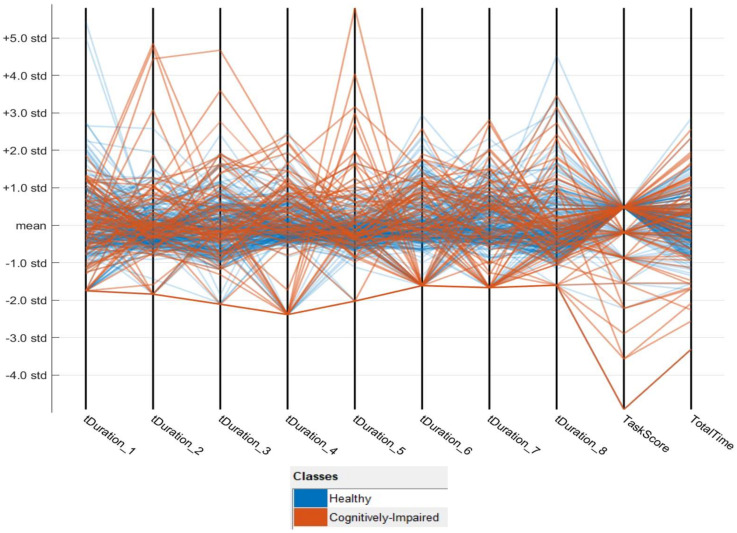
Parallel coordinates plot of cognitively impaired vs healthy using standard deviation.

In the initial dataset, a total of 35~59 features are extracted. However, one of the focus in this research is more on extracting the features that would be common to most of the scenarios in the living settings of older adults. Hence, the time duration features of IADLs such as tDuration_# (# means task number 1, 2, …, 3 etc.) and few others such as task completion score, total sensor event counter, etc. are considered to impact greatly on the outcome of the model. The parallel coordinates plot shows the overview of multi-dimensional data in a 2D patterns of the data, as shown in Figure 5. This plot shows fundamental relationships between features and can be used to identify useful predictors for separating healthy and impaired classes. It can be identified that the ‘tDuration_5′ and ‘TaskScore’ parameter containing the most variation of the class identifier.

It is mentioned earlier that when a user does not perform a task, the system puts task duration as zero, which is referred to as a missing value in the dataset. However, it should provide a considerable value to indicate the IADL is not completed. Zero seconds may indicate that a task is done very quickly, which may negatively influence the model. While the data is plotted via Parallel coordinates plot, it shows the task duration of zero second does not provide a reasonable deviation from the other data points. Therefore, the missing values are replaced of the duration using the following algorithm that will give a better standard deviation.
**Algorithm 2** Procedure to replace missing valuesStart of Loop, For each missing values of task durations (tDuration_1 to tDuration_8) Step 1. tDuration = Column name of the missing value Step 2. Category = category of the missing value (Cognitively-Impaired or Cognitively-Healthy) Step 3. MaxValue = Maximum value from the set [Category, tDuration] Step 4. Replace the missing value with two times of “MaxValue”.Continue the loop till the last missing value of the task durations. Step 5. Re-calculate the “TotalTime” parameter with the updated values from tDuration_1 to tDuration_8

**Figure 6:**
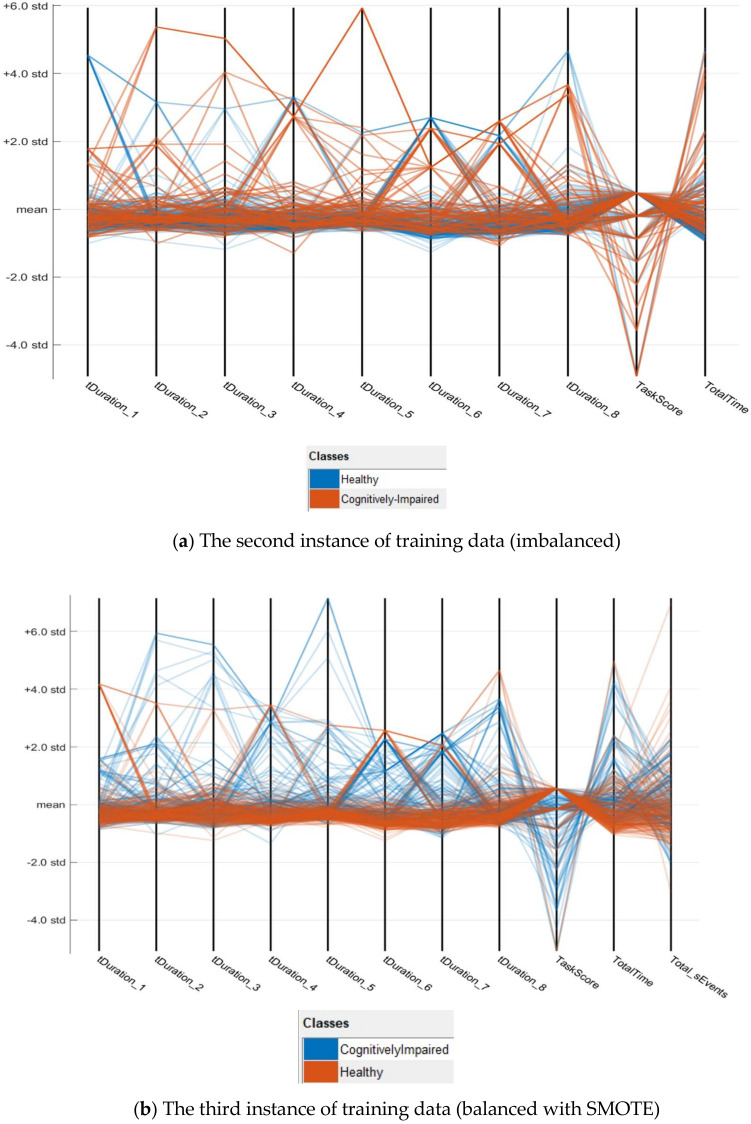
Parallel coordinates plots of cognitively impaired vs. healthy using standard deviation after replacing the missing values. (**a**) The second instance of training data (imbalanced) (**b**) The third instance of training data (balanced with SMOTE)

After the dataset is fixed with missing values as described in Algorithm 2, there are 76 records of impaired class and cognitively healthy class has 228 records. In the previous section, it is already mentioned how the data is divided into training and testing. On the third instance of training data SMOTE algorithm is applied to increase the records of minority class to balance the dataset. From the training sets of data, two more parallel coordinates plots are created. These plots provide an overview of multi-dimensional data on a single plot to see 2D patterns of the data as per Figure 6a,b. There are some differences among Figure 5 and Figure 6a,b. Especially in Figure 6a,b, the data points of cognitively healthy people are more consistent and stable. The outliers are mostly the cognitively impaired. Therefore, it is correct to put a considerable value as per algorithm#2 in the time duration of each task in the dataset when a task is not done.

**Figure 7:**
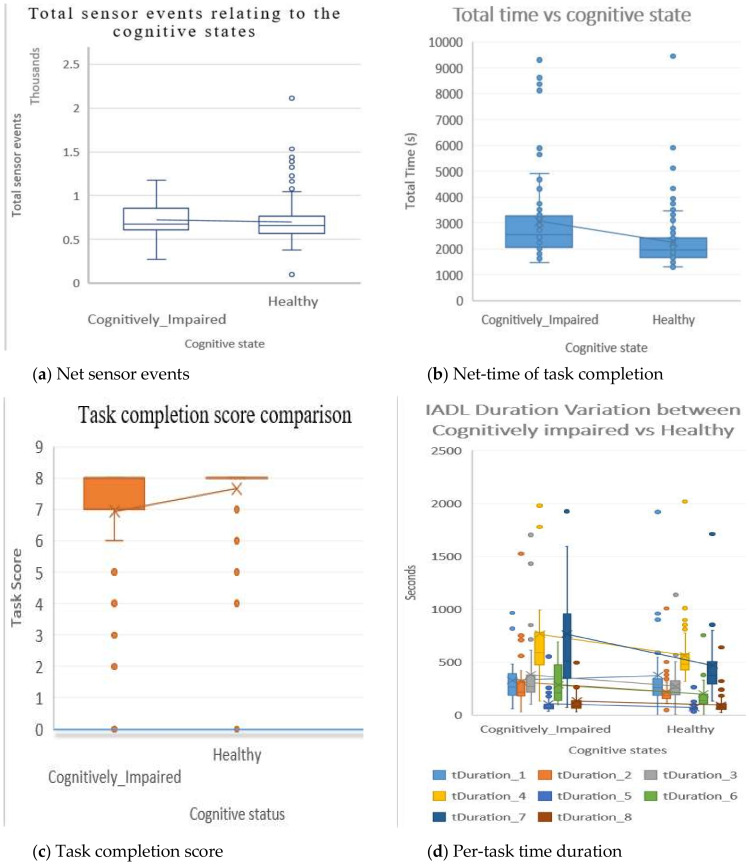
Patterns in the dataset on various parameters. (**a**) Net sensor events; (**b**) Net-time of task completion (**c**) Task completion score (**d**) Per-task time duration

Using the box and whisker chart, in Figure 7, the second instance of the dataset can be visualised into quartiles, highlighting the mean and outliers. Figure 7a shows the diversity of sensor events counter in cognitively impaired vs healthy cohort. The mean of total sensor events is very close between cognitively impaired and healthy participants. Although the mean value of the sensor events of these two groups is very close to each other, net task completion time has modest differences between the two cohorts. The healthy group could complete all the tasks quicker than the cognitively impaired group. Therefore, this parameter may contribute more to identify the onset of cognitive impairment. In Figure 7, the boxes have lines extending vertically, generally called “whiskers”. These lines in the box indicate variability outside the upper and lower quartiles of the data. There are some points outside those lines or whiskers that are considered an outlier for both cognitively impaired group and healthy group. Figure 7b shows the mean difference of the time on the completion of all assigned tasks. It can be identified that cognitively impaired individuals require more time to complete the tasks.

As shown in Figure 7c, most of the cognitively healthy participants completed all the assigned IADLs comparing with cognitively impaired ones missing some regular tasks. Cognitively impaired persons scored little less on task completion, including a few scorings very low. There is an increased number of outlier data on the cognitive impaired group to complete IADLs as compared to the healthy group. In Figure 7d, the per-task time duration is plotted between the two groups. From the plot, there are “tDuration_7” and “tDuration_4” features demonstrating significant mean differences between the healthy and cognitively impaired groups. In the next section, the ranking of the parameters is analysed within the dataset to identify the two categories.

### 5.2. Feature Ranking

Here, the features are evaluated which are relevant and essential to distinguish the data into the correct categories. Features are ranked for classification using the Minimum Redundancy Maximum Relevance (MRMR) algorithm [49]. This algorithm measures mutual information between two variables. It calculates how much uncertainty of one variable can be reduced by calculating the relationship with the other variable. The mutual information *E* of the discrete random variables X and Z is defined by the following equation.

(2) $$E\left(X,Z\right)=\;\sum_{i,j}P\left(X=x_{i},\;Z=z_{j}\right)log\frac{P\left(X=x_{i},\;Z=z_{j}\right)}{P\left(X=x_{i})P(Z=z_{j}\right)}$$

*E* equals 0 when *X* and *Z* are independent. On the other hand, *E* equals the entropy of *X* when *X* and *Z* are the same random variable.

The MRMR algorithm finds an optimal set of features that are mutually and maximally dissimilar and can represent the healthy class and cognitively class effectively. The algorithm minimises the redundancy of a feature set and maximising the relevance of a feature set to the response variable. The goal of the MRMR is to find an optimal set *S* of features that maximises *V_S_*, the relevance of *S* concerning a response variable y, but minimises *W_S_*, the redundancy of *S* where *V_S_* and *W_S_* are defined with mutual information *E*

(3) $$V_{s}=\frac{1}{\left|S\right|}\sum_{x\in S}E\left(x,y\right)$$

(4) $$W_{s}=\frac{1}{{\left|S\right|}^{2}}\sum_{x,z\in S}E\left(x,z\right)$$

where |*S*| is the number of predictors in *S*.

Finding an optimal set *S* requires considering all 2^|Ω|^ combinations, where Ω is the entire feature set. Instead, the MRMR ranks predictors through the forward addition scheme, which requires O(|Ω|·|*S*|) computations, by using the mutual information quotient (MIQ) value, which is defined as follows:

(5) $$MIQ_{x}=\frac{V_{x}}{W_{x}}$$

where *V_x_* and *W_x_* are the relevance and redundancy of a predictor, respectively as below:

(6) $$V_{x}=E\left(x,y\right)$$

(7) $$W_{x}=\frac{1}{\left|S\right|}\sum_{z\in S}E\left(x,z\right)$$

**Figure 8:**
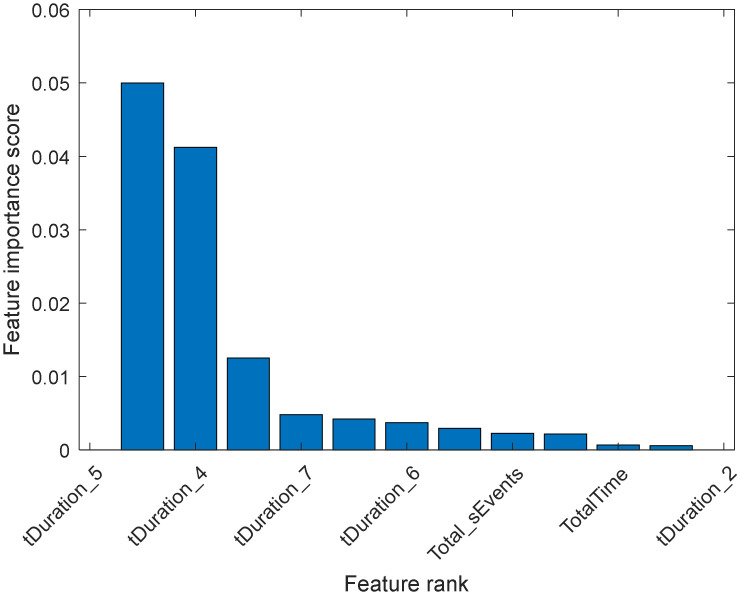
Feature ranking amongst pre-selected features.

The importance score represents how much a feature influences the output in the dataset. When only the pre-selected features of time duration are used, and task completion score and total sensor events score, the drop in score between the second and third most important features is significant. In contrast, the slides after the 4th predictor are relatively small. A decrease in the importance score represents the confidence of features selected. Therefore, the significant reduction implies that there is more confidence in choosing the more important predictor. The small drops indicate that the difference in predictor importance is not substantial. After the MRMR algorithm is applied to the selected features, the results of their ranking are shown in Figure 8. In Figure 8, “tDuration_5” in the dataset is the most important predictor of the dataset among the pre-selected features, followed by “tDuration_4”, “tDuration_7”, “tDuration_6” and so on.

**Figure 9:**
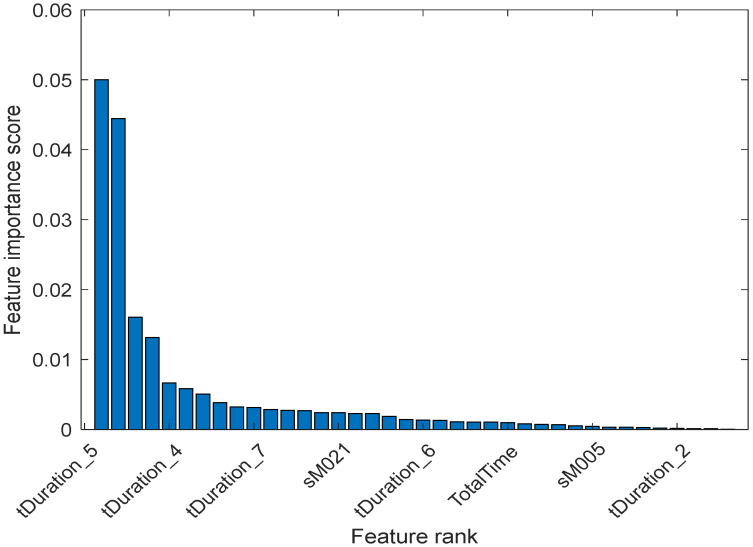
All the feature ranking based on importance score.

Alternatively, if all the 39 features are considered from the dataset instead of 12 features used in Figure 8, a slightly different ranking has resulted, as shown in Figure 9. MRMR algorithm is used to rank all the extracted features, regardless of and predefined usability of the features.

In Figure 9, the drops in score between the second and third most important features are significant, while the slides after the 9th feature are relatively small. A reduction in the important score means less confidence in feature or predictor selection for those features. In Figure 9, the feature importance score can be observed. From the result, the “tDuration_5” in the dataset should be the most important predictor. Two sensors “M021” and “M005” are ranked more important when all the 39 features are included. In a real-world deployment, it is not possible to place a sensor at an exact position all the time. Therefore, it is essential to focus on to create a more flexible and generic model. The generic model should not be biased regarding the total number and location of the motion sensors. Therefore, the only total number of motion sensors events are included in the next section to build the model.

Additionally, in the second training instance, another feature is added based on the participant’s age to investigate the impact on the accuracy of the model. The average age and age range of the participants are mentioned in the dataset. Hence, a new feature based on participants age is included in the second training instance.

Here is the list of the top features based on MRMR rank:

1. ‘tDuration_5’
2. ‘tDuration_4’
3. ‘tDuration_7’
4. ‘sM021’
5. ‘tDuration_6’
6. ‘TotalTime’
7. ‘sM005’
8. ‘tDuration_2’
9. ‘TaskScore’
10. ‘tDuration_8’
11. ‘tDuration_3’
12. ‘tDuration_1’
13. ‘Total_sEvents’

From the rank above, the duration of the ADLs (tDuration_#) are the most important indicators, including the net time (TotalTime), task score (TaskScore), and the total number of sensor events (Total_sEvents). These properties of the data match with the presumption that there could be noticeable differences to complete a given task or daily activities by the cognitive impaired person compare to a healthy person. Considering this information, in the next section, several models are going to be built and will be tested with the selected features. These selected features are determined from the ranked list as these are identified to be important to classify the onset of dementia.

## 6. Model Development and Performance Analysis

In this section, several comparative models are built to identify the onset of dementia. To determine which classification techniques to be used in the final model, the performance of the different classification techniques are evaluated, including the K-fold cross-validation method with several machine learning classifier algorithms. The target model should be able to advise the older adults to see a doctor if they are showing early symptoms. Hence, bias was added on some occasions to increase the true positive rate and to decrease false negative to identify the onset of dementia.

### 6.1. Model Building and Training Performance

The dataset is labelled, which means it provides the data as well as classification of the data—either each sample is under a healthy class or cognitively impaired class. Furthermore, the dataset is not very large; therefore, a supervised machine learning method is applied, and 5-fold cross-validation is used to train the classification models that are mentioned in Table 2. Furthermore, the FFNN based model is also developed and trained to compare with the classifiers.

Several classification methods are tested to compare which one is suitable to provide an excellent model to identify cognitive impairment. A lower number of K-fold cross-validation and RUSBoosted algorithm can reach an optimal and less erroneous model for imbalanced data. Hence, 5-fold Cross-Validation is applied, and the performance of the algorithms is compared, as shown in Table 2. The classifiers have used the imbalanced dataset for the initial training. FFNN is trained with balanced dataset duplicated values of the minority class, as mentioned earlier. In Table 2, TN represents True Negative; FN stands for False Negative, FP as False Positive and TP as True Positive. F-Score is the harmonic mean of precision and sensitivity, F-Score can be calculated as below,

(8) $$F-Score=\frac{2TP}{2TP+FP+FN}$$

From Table 2, it is visible that Kernel-based Naïve Bayes and RUSBoosted Ensemble model stands out, including FFNN model, which has F-Score 83.73%. Next, to improve and optimise the model, a custom misclassification matrix is used to increase TP values and decrease FN values. Table 3 shows the model training performance after FN cost is increased by 30. The last column provides total misclassification cost.

It can be observed from Table 3, Decision tree, Naïve Bayes and RUSBoosted Ensemble model provided better performance. Naïve Bayes has the best F-Score of 78.81%. Additionally, Table 4 represents the model training performance with SMOTE generated balanced dataset. Here, Cubic KNN provides 94.92% precision and Ensemble Bagged Tree achieves 91.60% F-score. However, after the SMOTE process, FFNN is showing weakness in the training model, reaching 75.46% F-score.

In the next section, the better performing models will be tested further with test dataset, and this analysis will assist in deciding to best model to deploy.

### 6.2. Testing Performance

The test dataset contains data samples of the cognitively impaired and healthy person. The test dataset contains 54 data points. The performance of the models from Table 3 based on the test dataset is showing in Table 5. It can be observed that RUSBoosted Ensemble model is very accurate with 92.59% accuracy where FFNN achieved highest F-Score as 91.89%. The Median Tree shows 100% precision in identifying the cognitively impaired subjects. However, it significantly failed to reach an optimal F-Score. FFNN model is built duplicated minor class data in the training period, and it showed optimal performance on the test samples. The other models did perform well during the training phase—however, those performed poorly on the test dataset. For example, from Table 5 it can be observed that the Naïve Bayes model had a good score in the training phase Table 3 and Table 4. However, on the testing dataset, this model did not perform well. The SVM and KNN models have also demonstrated poor performance on the test dataset.

Table 6 contains the performance result of the test dataset of the models that are created in Table 4. Fine Decision Tree provides best F-Score of 90.58%; Ensemble Boosted Tree has zero FN value that is helpful to ensure most of the suspected cases will be reported. However, RUSBoosted is most precise in finding early signs of cognitive impairment with a precision value of 90.00%.

### 6.3. Discussion

Based on the analysis from the result of Table 2, Table 3 and Table 5 Ensemble RUSBoosted Model and FFNN provide the best possible model with F-Score 88.89% and 90.67%. However, RUSBoosted has 100% precision on test data which is much better than FFNN, which has precision 65%. Considering imbalance dataset used in Ensemble classifier, this model is the right candidate for imbalanced dataset models.

On the other hand, after the SMOTE process dataset is balanced by increasing minor class data samples. The models that are created using SMOTE training dataset are from Table 4 and Table 6. It can be observed, Boosted Tree Ensemble provides 100% sensitivity and 88.52% F-score, Fine Decision Tree provides 90.74% accuracy, RUSBoosted Ensemble provides 90% precision, and Fine KNN provides 94.92% precision on training data and 80.99% F-score in testing data. Interestingly, the FFNN model did not perform well. Therefore, it should be disregarded for solving this problem. It is mentioned earlier that RUSBoosted algorithm can reach an optimal and less erroneous model for imbalanced data. Moreover, after balancing the dataset with SMOTE process, this algorithm also shows strong performance. FFNN works better when the size of the data sample is very large and can be used for developing a model. However, particularly in this problem, the data sample size is limited. Hence, the neural net-based model is not very reliable as it also appears in the result of the balanced dataset.

Hence, based on the F-score, Fine decision tree, Fine KNN and Ensemble boosted and RUSBoosted models are appropriate candidates to identify the onset of dementia using machine learning and IoT in a residential care setting.

### 6.4. Limitation

This work has focused on developing a model based on a new approach to detect the onset of dementia. It and combines related works to capture the start-time and end-time of IADL activity in a residential setting. The dataset only includes monitoring data of several hours in a uniform residential space. Hence, all the feature values are limited to the environment of the experiment. Furthermore, there are other complementary signs of the onset of dementia that are related to emotion and mood changes from repetitive speech, sleeping routine, screen time, etc. Those models can also be integrated with this model to provide a multimodal predictive suggestion. Security and privacy concerns of the IoT data is not included in this work as biometric token-based IoT and information security can be introduced as future work to ensure privacy and security of the older adults.

## 7. Conclusions

We have developed a model to identify the onset of dementia from the CASAS dataset. From the dataset, the important features are identified, such as total sensory events, the duration of completing ADLs, the total number of the completed task and other important features. These features contributed to developing the model to monitor the older adults remotely to identify the onset of dementia. The developed model can identify the onset of dementia, with 90.74% accuracy with the Decision Tree model compare to 88% accuracy in the previous work on the dataset. Overall, the RUSBoosed Ensemble-based model has provided optimal result for the imbalanced dataset and balanced dataset by reaching F-Score 88.89% and 87.38% respectively. Furthermore, in the previous work on this dataset over 350 features were considered to develop a model compare to below 50 features are used in this work, yet we have achieved better F-score and accuracy. Successfully predicting cognitive impairment of the person with dementia will further assist in providing predictive care for older adults. Hence, this work contributes to their enhanced independent living and their cognitive well-being. This result can be considered for older adults to advocate seeing a doctor for the cognitive anomaly. In our future works, we plan to deploy this model with selected features and validate with other datasets to observe the model behaviour and possible integration with the other models using transform learning and multimodal predictive process.

## Figures and Tables

**Table 1:** Activities performed by the participants.

No.	Activities	Tag Name
1.	Sweep the kitchen and dust the living room.	Task 1
2.	Obtain a set of medicines and fill a weekly medicine dispenser.	Task 2
3.	Write a birthday card, enclose a check, and address an envelope.	Task 3
4.	Find the appropriate DVD and watch the corresponding news clip.	Task 4
5.	Obtain a watering can and water all plants in the living space.	Task 5
6.	Answer the phone and respond to questions of the video from task 4.	Task 6
7.	Prepare a cup of soup using the microwave.	Task 7
8.	Pick a complete outfit for an interview from a selection of clothing.	Task 8

**Table 2:** Train the model with Imbalanced Data (Except FFNN).

Cognitively Impaired Class									
Model Type	TN	FN	FP	TP	Sensitivity	Specificity	Precision	Accuracy	F-Score
Coarse Decision Tree	169	25	25	31	55.36%	87.11%	55.36%	80.00%	67.70%
Linear Discriminant	185	42	9	14	25.00%	95.36%	60.87%	79.60%	39.61%
Logistics Regression	184	39	10	17	30.36%	94.85%	62.96%	80.40%	45.99%
Kernel Naïve Bayes	178	18	16	38	67.86%	91.75%	70.37%	86.40%	78.02%
Quadratic SVM	182	30	12	26	46.43%	93.81%	68.42%	83.20%	62.12%
Cubic KNN	186	39	8	17	30.36%	95.88%	68.00%	81.20%	46.11%
Ensemble Bagged Trees	187	30	7	26	46.43%	96.39%	78.79%	85.20%	62.67%
Ensemble RUSBoosted	155	14	39	42	75.00%	79.90%	51.85%	78.80%	77.37%
FFNN	175	19	29	177	90.31%	85.78%	85.92%	88.00%	87.99%

**Table 3:** Train the model with a bias to reduce FN and increase TP value.

Cognitively Impaired Class (Imbalanced Data)										
Model Type	TN	FN	FP	TP	Sensitivity	Specificity	Precision	Accuracy	F-Score	Cost
Fine Decision Tree	159	14	35	42	75.00%	81.96%	54.55%	80.40%	78.33%	455
Discriminant Linear	59	2	135	54	96.43%	30.41%	28.57%	45.20%	46.24%	175
Naïve Bayes Kernel	150	11	44	45	80.36%	77.32%	50.56%	78.00%	78.81%	264
SVM Quadratic	133	21	61	35	62.50%	68.56%	36.46%	67.20%	65.39%	481
KNN Cubic	173	30	21	26	46.43%	89.18%	55.32%	79.60%	61.06%	621
Ensemble RUSBoosted	158	14	36	42	75.00%	81.44%	53.85%	80.00%	78.09%	456

**Table 4:** Train the model with balanced data using SMOTE.

Cognitively Impaired									
Model Type	TN	FN	FP	TP	Sensitivity	Specificity	Precision	Accuracy	F-Score
Fine Decision Tree	158	36	24	173	82.78%	86.81%	87.82%	84.65%	84.75%
Linear Discriminant	147	47	86	111	70.25%	63.09%	56.35%	65.98%	66.48%
Logistics Regression	147	47	81	116	71.17%	64.47%	58.88%	67.26%	67.65%
Kernel Naïve Bayes	157	37	52	145	79.67%	75.12%	73.60%	77.24%	77.33%
Fine Gaussian SVM	144	50	15	182	78.45%	90.57%	92.39%	83.38%	84.07%
Fine KNN	149	45	10	187	80.60%	93.71%	94.92%	85.93%	86.66%
Ensemble Boosted Tree	174	20	13	184	90.20%	93.05%	93.40%	91.56%	91.60%
Ensemble RUSBoosted	155	39	19	178	82.03%	89.08%	90.36%	85.17%	85.41%
FFNN	149	45	51	146	76.44%	74.50%	74.11%	75.45%	75.46%

**Table 5:** Testing the models.

Cognitively Impaired									
Model Type	TN	FN	FP	TP	Sensitivity	Specificity	Precision	Accuracy	F-Score
Median Tree	34	16	0	6	27.27%	100.00%	100.00%	71.43%	42.86%
Kernel Naïve Bayes	33	11	1	9	45.00%	97.06%	90.00%	77.78%	61.49%
SVM	33	13	1	7	35.00%	97.06%	87.50%	74.07%	51.45%
KNN	31	14	3	6	30.00%	91.18%	66.67%	68.52%	45.15%
RUSBoosted Ensemble	34	4	0	16	80.00%	100.00%	100.00%	92.59%	88.89%
FFNN	34	0	6	14	100.00%	85.00%	70.00%	88.89%	91.89%

**Table 6:** Testing the models created from SMOTE data.

Cognitively Impaired									
Model Type	TN	FN	FP	TP	Sensitivity	Specificity	Precision	Accuracy	F-Score
Fine Decision Tree	31	2	3	18	90.00%	91.18%	85.71%	90.74%	90.58%
Kernel Naïve Bayes	29	6	5	14	70.00%	85.29%	73.68%	79.63%	76.89%
Fine Gaussian SVM	27	1	5	19	95.00%	84.38%	79.17%	88.46%	89.37%
Fine KNN	24	1	10	19	95.00%	70.59%	65.52%	79.63%	80.99%
Boosted Tree Ensemble	27	0	7	20	100.00%	79.41%	74.07%	87.04%	88.52%
RUSBoosted Ensemble	30	4	2	18	81.82%	93.75%	90.00%	88.89%	87.38%
FFNN	28	6	6	14	70.00%	82.35%	70.00%	77.78%	75.68%
